# Hookworm: “The Great Infection of Mankind”

**DOI:** 10.1371/journal.pmed.0020067

**Published:** 2005-03-29

**Authors:** Peter J Hotez, Jeff Bethony, Maria Elena Bottazzi, Simon Brooker, Paulo Buss

## Abstract

Over the last five years, there has been increasing recognition of the global health importance of hookworm. New international efforts to control the morbidity of hookworm are in progress

## Introduction

In 1962, Norman Stoll, the distinguished Rockefeller Institute scientist who helped to establish human parasitology research in North America, described the unique health impact of hookworm as follows [1]:

As it was when I first saw it, so it is now, one of the most evil of infections. Not with dramatic pathology as are filariasis, or schistosomiasis, but with damage silent and insidious. Now that malaria is being pushed back hookworm remains the great infection of mankind. In my view it outranks all other worm infections of man combined…in its production, frequently unrealized, of human misery, debility, and inefficiency in the tropics.

Like many other global disease experts who witnessed dramatic reductions in malaria prevalence as a result of DDT spraying during the late 1950s [2], Stoll did not anticipate malaria's imminent re-emergence in India. However, he articulated with eloquence the magnitude of the disease burden resulting from hookworm infection. He further offered the silent and insidious character of hookworm as a partial explanation for its neglect by the global medical community.

This neglect subsequently intensified during the 1970s, 1980s, and 1990s with the omission of hookworm from the list of diseases covered by the World Health Organization's Special Programme for Research and Training in Tropical

Hookworm has proven to be extremely difficult to eliminate or eradicate in areas of poverty and poor sanitation.

Diseases, as well as from other global health initiatives. Over the last ten years, however, there has been increasing recognition of the global health importance of hookworm. Today, new international efforts to control the morbidity of hookworm and other soil-transmitted helminth infections are in progress (www.who.int/wormcontrol).

## Etiology and Global Distribution

Human hookworm infection is caused by blood-feeding nematode parasites of the genus Ancylostoma and the species Necator americanus. Worldwide, N. americanus is the predominant etiology of human hookworm infection, whereas A. duodenale occurs in more scattered focal environments [3]. These two hookworms, together with the roundworm, Ascaris lumbricoides, and the whipworm, Trichuris trichiura, are often referred to collectively as soil-transmitted helminths (STHs).

**Figure 1:**
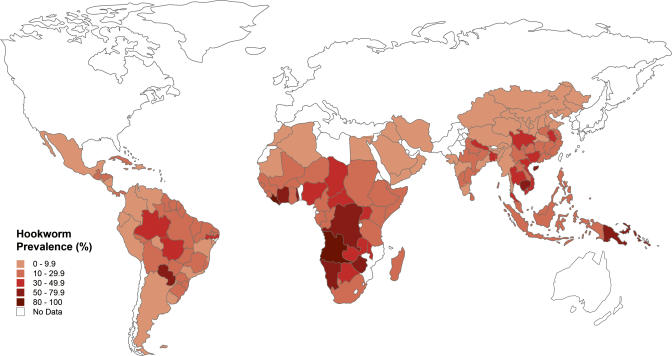
Global Distribution of Human Hookworm Infection (Illustration: Margaret Shear, Public Library of Science, adapted from [4])

**Figure 2:**
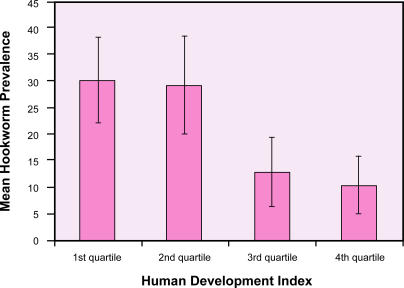
The Relationship between Poverty and Hookworm Prevalence (Illustration: Margaret Shear, Public Library of Science, adapted from [4])

No international surveillance mechanisms are in place to determine the prevalence and global distribution of hookworm infection. However, based on an extensive search of the literature since 1990, the worldwide number of cases of hookworm was recently estimated to be 740 million people [4]. The highest prevalence of hookworm occurs in sub-Saharan Africa and eastern Asia (Figure 1). High transmission (defined below) also occurs in other areas of rural poverty in the tropics, including southern China [5], the Indian subcontinent [6], and the Americas [7]. In all regions, there is a striking relationship between hookworm prevalence and low socioeconomic status (Figure 2) [4]. Hookworm's neglected status partly reflects its concentration among the world's poorest 2.7 billion people who live on less than $2 a day.

## Clinical Features, Epidemiology, and Disease Burden

**Figure 3:**
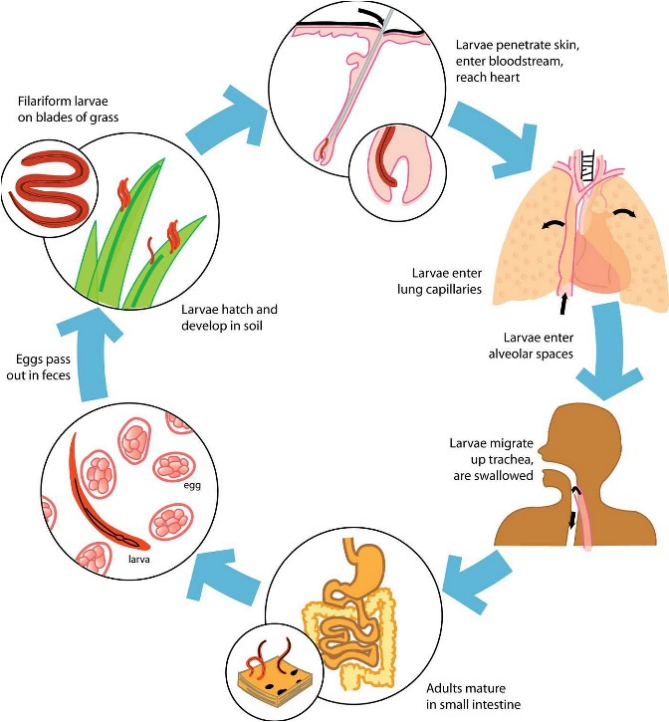
Life Cycle of the Human Hookworm N. americanus The BZA anthelminthics albendazole and mebendazole remove adult hookworms from the gastrointestinal tract. In contrast, the *Na*-ASP-2 Hoookworm Vaccine is designed to target third-stage infective larvae (filariform larvae). Humoral immunity to the vaccine inhibits the entry of larvae into the gastrointestinal tract and thereby prevents their development into blood-feeding adult parasites. (Illustration: Sapna Khandwala, Public Library of Science, adapted from [3] and [33])

Hookworm infection is acquired by invasion of the infective larval stages through the skin (A. duodenale larvae are also orally infective). Following host entry, the larvae undergo a journey through the vasculature, then the lungs and other tissues, before they enter the gastrointestinal tract and molt twice to become one-centimeter-long adult male and female worms [3]. The worms mate and the female hookworms produce up to 30,000 eggs per day, which exit the host's body in the feces (Figure 3).

Because hookworms do not replicate in humans, the morbidity of hookworm is highest among patients that harbor large numbers of adult parasites. Estimates of the intensity of hookworm infection are typically obtained by using quantitative fecal egg counts as a surrogate marker for worm burden. The World Health Organization defines moderate-intensity infections as those with 2,000–3,999 eggs per gram of feces, and heavy-intensity infections as those with 4,000 or more eggs per gram (p. 26 in [8]). Compared to other STH infections and schistosomiasis, hookworm infection exhibits a unique age-intensity profile—whereas the intensities for the former peak in childhood and adolescence, hookworm intensity usually either steadily rises in intensity with age or plateaus in adulthood [3,9]. The biological basis for this observation is unknown [10].

Adult hookworms cause morbidity in the host by producing intestinal hemorrhage [3]. The adult hookworms then ingest the blood, rupture the erythrocytes, and degrade the hemoglobin [11]. Therefore, the disease attributed to hookworm is silent blood loss leading to iron deficiency anemia and protein malnutrition. There is a correlation between parasite intensity and host intestinal blood loss [12]; in children, women of reproductive age, and other populations with low iron stores, there is often a correlation between parasite intensity and reductions in host hemoglobin [3,12,13,14,15,16]. In children, chronic heavy-intensity infections are associated with growth retardation, as well as intellectual and cognitive impairments; in pregnant women, they are associated with adverse maternal–fetal outcomes [3,12,13,14,15,16].

When measured in disability-adjusted life years, the global disease burden from hookworm exceeds all other major tropical infectious diseases with the exception of malaria, leishmaniasis, and lymphatic filariasis (pp. 192–193 in [17]). In addition, hookworm has been associated with impaired learning, increased absences from school, and decreased future economic productivity [18]. Therefore, like other neglected diseases, chronic infection with hookworm promotes long-term disability and increases the likelihood that an afflicted population will remain mired in poverty.

## Hookworm Control Strategies

Because of its high transmission potential, hookworm has proven to be extremely difficult to eliminate or eradicate in areas of poverty and poor sanitation [19]. Indeed, in the absence of comprehensive economic development, the impact of sanitation, footwear, and health education has been minimal [19]. Control efforts have therefore shifted to reducing morbidity through mass treatment (also known as “deworming”) of affected populations with anthelminthic drugs [19].

Although benzimidazoles (BZAs) are the most commonly used agents for treating STH infections, levamisole and pyrantel may also be used in some circumstances. Periodic and repeated deworming with BZAs and praziquantel, complemented by basic sanitation and adequate safe water, is considered the most cost-effective means to control the morbidity caused by STH and schistosome infections [19,20,21,22]. Efforts led by the World Health Organization have focused on annual, twice-yearly, or thrice-yearly doses in schools because the heaviest intensities of STH infections are most commonly encountered in school-age children [23].

Among the health benefits of periodic deworming of schoolchildren are improvements in iron and hemoglobin status, physical growth and fitness, and cognition [20,21,22,23]. In addition, there are important externalities, including improvements in education and reduced community-based transmission of ascaris and trichuris infections [23]. Accordingly, at the 54th World Health Assembly in 2001, a resolution was passed urging member states to attain a minimum target of regular deworming of at least 75% and up to 100% of all at-risk school-age children by 2010 [20,23].

## Developing a New Control Tool: The *Na*-ASP-2 Hookworm Vaccine

Deworming satisfies a number of United Nations Millennium Development Goals including those related to poverty reduction, child health, and education. However, there are also several reasons to believe that the school-based deworming programs could have less of an impact on the control of morbidity from hookworm than from other STH and schistosome infections [3]. As noted above, heavy-intensity hookworm infections are common among both adults and children, so school-based programs would not be expected to have an impact on hookworm transmission in the community [24]. School-based programs are also not likely to affect either preschool children or pregnant women, despite evidence for the health benefits from BZAs in both populations [16,25]. Finally, a single dose of mebendazole (one of the two major BZAs) has variable efficacy against hookworm [26], and following treatment, hookworm reinfection to pre-treatment levels can occur within 4–12 months [27]. This, and the observation that the efficacy of mebendazole against hookworm can diminish with frequent and repeated use, has prompted concerns about the possible emergence of BZA resistance [28].

As a complementary strategy, the Human Hookworm Vaccine Initiative (HHVI) is developing a safe, efficacious, and cost-effective vaccine, the *Na*-ASP-2 Hookworm Vaccine, that would provide an additional tool for the control of hookworm [29,30]. The HHVI is a non-profit partnership comprising research, process development, vaccine manufacturing and control, and pre-clinical and clinical testing units at the George Washington University, London School of Hygiene and Tropical Medicine, and Oswaldo Cruz Foundation (FIOCRUZ), and sponsored by the Sabin Vaccine Institute (www.sabin.org).

The HHVI selected the hookworm larval antigen ASP-2 (ancylostoma secreted protein-2) based on studies that (1) identified the molecule as a protective antigen linked to earlier-generation irradiated infective larval vaccines [29], (2) determined a relationship between human anti-ASP-2 antibodies and reduced risk of heavy hookworm infection in populations living in hookworm-endemic regions of Brazil and China ([30]; J. Bethony, A. Loukas, M. J. Smout, S. Brooker, S. Mendez, et al., unpublished data), and (3) confirmed the ability of recombinant ASP-2 to partially protect laboratory animals against larval hookworm challenges [30,31,32].

**Figure 4:**
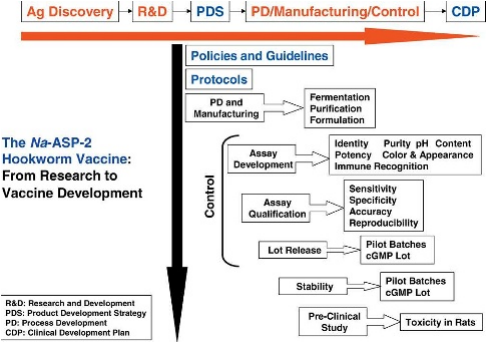
Scheme for the Development and Quality-Control Testing of the *Na*-ASP-2 Hookworm Vaccine, and Its Transition from the Laboratory into the Clinic After the selection of ASP-2 from N. americanus (*Na*-ASP-2) as the lead candidate antigen based on a series of research and development (R&D) tests—which included immunoepidemiology studies identifying human correlates of immunity to hookworm and confirmatory laboratory animal vaccine trials—the recombinant antigen was expressed in yeast and then developed as a biologic through a well-defined product development strategy (PDS). By following the product development strategy, process development (PD) and manufacturing led to the generation of pilot batches at different scales prior to technology transfer to a cGMP manufacturing facility. Both process development and manufacturing rely on developing assays for the product's identity, color and appearance, purity, immunological recognition, and potency, as well as qualification of the assays for sensitivity, specificity, accuracy, and reproducibility. Each of these processes must maintain a high level of quality control by following a set of policies, protocols, and standard operating procedures. After the manufacturing of a cGMP product and the required pre-clinical animal testing, a clinical development plan (CDP) was generated. Because the *Na*-ASP-2 Hookworm Vaccine is a product destined for the world's poorest, it is being developed almost exclusively in the non-profit sector, along with government manufacturers in middle-income countries.

Process development, cGMP manufacture and control, and pre-clinical testing of *Na*-ASP-2 from N. americanus were completed in 2004 (Figure 4). Pending United States Food and Drug Administration approval, clinical testing of the vaccine will take place in 2005. The *Na*-ASP-2 Hookworm Vaccine will be developed almost entirely in the non-profit sector. Ultimately, the vaccine will be indicated for the active immunization of susceptible individuals against moderate and heavy necator infection. Vaccination would reduce the number of hookworm infective larvae entering the gastrointestinal tract, thereby reducing the number of adult worms and the fecal egg counts in individuals exposed to the larvae.

## Hookworm as a Model

Because immunization would only affect hookworm larvae and not adult hookworms already residing in the gastrointestinal tract of infected individuals, the first dose of the vaccine would be administered following deworming. Therefore, use of the vaccine could build on the infrastructures developed as part of school-based programs. Given that hookworm afflicts only the world's most impoverished, a major hurdle for the development of the *Na*-ASP-2 Hookworm Vaccine is its small commercial market. Innovative financing mechanisms must be considered to produce this orphan biologic. Towards that end, the HHVI has partnered with manufacturers in hookworm-endemic middle-income countries that would commit to industrial scale-up of the *Na*-ASP-2 Hookworm Vaccine pending proof-of-principle for its efficacy. This approach might help to inform the development of business models for the production and distribution of orphan biologics for other neglected diseases.

## Abbreviations

BZA: benzimidazole
HHVI: Human Hookworm Vaccine Initiative
STH: soil-transmitted helminth
